# Individual and joint contributions of genetic and methylation risk scores for enhancing lung cancer risk stratification: data from a population-based cohort in Germany

**DOI:** 10.1186/s13148-020-00872-y

**Published:** 2020-06-18

**Authors:** Haixin Yu, Janhavi R. Raut, Ben Schöttker, Bernd Holleczek, Yan Zhang, Hermann Brenner

**Affiliations:** 1 Division of Clinical Epidemiology and Aging Research, German Cancer Research Center (DKFZ), Im Neuenheimer Feld 581, 69120 Heidelberg, Germany; 2 Medical Faculty Heidelberg, University of Heidelberg, Im Neuenheimer Feld 672, 69120 Heidelberg, Germany; 3 Division of Preventive Oncology, German Cancer Research Center (DKFZ) and National Center for Tumor Diseases (NCT), Im Neuenheimer Feld 460, 69120 Heidelberg, Germany; 4 Network Aging Research, University of Heidelberg, Bergheimer Straße 20, 69115 Heidelberg, Germany; 5 Saarland Cancer Registry, Krebsregister Saarland, Präsident-Baltz-Straße 5, 66119 Saarbrücken, Germany; 6 German Cancer Consortium (DKTK), German Cancer Research Center (DKFZ), Im Neuenheimer Feld 280, 69120 Heidelberg, Germany

**Keywords:** Lung cancer, Risk prediction, Polygenic risk score, DNA methylation

## Abstract

**Background:**

Risk stratification for lung cancer (LC) screening is so far mostly based on smoking history. This study aimed to assess if and to what extent such risk stratification could be enhanced by additional consideration of genetic risk scores (GRSs) and epigenetic risk scores defined by DNA methylation.

**Methods:**

We conducted a nested case-control study of 143 incident LC cases and 1460 LC-free controls within a prospective cohort of 9949 participants aged 50–75 years with 14-year follow-up. Lifetime smoking history was obtained in detail at recruitment. We built a GRS based on 31 previously identified LC-associated single-nucleotide polymorphisms (SNPs) and a DNA methylation score (MRS) based on methylation of 151 previously identified smoking-associated cytosine-phosphate-guanine (CpG) loci. We evaluated associations of GRS and MRS with LC incidence by logistic regression models, controlling for age, sex, smoking status, and pack-years. We compared the predictive performance of models based on pack-years alone with models additionally including GRS and/or MRS using the area under the receiver operating characteristic curve (AUC), net reclassification improvement (NRI), and integrated discrimination improvement (IDI).

**Results:**

GRS and MRS showed moderate and strong associations with LC risk even after comprehensive adjustment for smoking history (adjusted odds ratio [95% CI] comparing highest with lowest quartile 1.93 [1.05–3.71] and 5.64 [2.13–17.03], respectively). Similar associations were also observed within the risk groups of ever and heavy smokers. Addition of GRS and MRS furthermore strongly enhanced LC prediction beyond prediction by pack-years (increase of optimism-corrected AUC among heavy smokers from 0.605 to 0.654, NRI 26.7%, *p* = 0.0106, IDI 3.35%, *p* = 0.0036), the increase being mostly attributable to the inclusion of MRS.

**Conclusions:**

Consideration of MRS, by itself or in combination with GRS, may strongly enhance LC risk stratification.

## Background

Lung cancer (LC) is the leading cause of cancer-related death worldwide, accounting for more than 1,761,000 deaths in 2018 [1]. Prognosis of LC is generally poor, with 5-year survival ranging from 10 to 20% in different countries [2]. Poor survival is due to the majority of tumors being detected at an advanced stage, at which options for curative treatment are limited [3–5]. Survival and prognosis may be much better when LC is detected at an early stage through the use of screening [6]. Randomized trials have demonstrated that the potential of reducing LC mortality by screening the high-risk group of heavy smokers with low-dose computed tomography (LDCT) [6, 7].

Risk stratification for LC screening is so far mostly based on smoking history [7]. Although smoking is a major risk factor for LC, less than 20% of lifelong smokers develop LC, and a non-negligible proportion of LCs also occur in people not meeting the definitions of heavy smoking [8]. Therefore, additional markers for pre-selecting those at highest risk for LC screening are highly desirable. In the past decade, dozens of single-nucleotide polymorphisms (SNPs) associated with LC risk have been identified through genome-wide association studies (GWAS) [9]. Genetic risk scores (GRS) based on SNPs identified from GWAS studies were found to enhance performance of risk prediction models for several common illnesses, such as cardiovascular disease [10], diabetes [11, 12], breast cancer [13, 14], colorectal cancer [15], and prostate cancer [16, 17]. However, few studies have evaluated the contributions of GRS to risk stratification of LC.

Studies have shown that changes in DNA methylation in blood prior to lung cancer diagnosis mainly occur at smoking-associated genes [18, 19]. In recent years, epigenome-wide association studies (EWAS) have identified a large number of CpG sites in whole blood DNA whose methylation levels were strongly related to smoking history and were also found to be related to LC risk [20, 21]. In this study, we aimed to assess the individual and joint potential of a GRS and a methylation risk score (MRS) based on smoking-related CpGs for enhancing LC risk stratification in a cohort of older adults who were followed for 14 years.

## Results

### Participant characteristics

The characteristics of the 143 LC cases and 1460 controls at baseline are presented in Table 1. The mean age was 63.7 years in cases and 61.8 years in controls. The proportion of males, current smokers, and especially heavy smokers was much higher among cases than among controls. The median GRS was 28 (range 18 to 42) and 27 (range 12 to 41) in cases and controls, respectively, and the median MRS was 0.78 (range − 0.35 to + 2.48) and 0.15 (range − 0.84 to + 3.47) in cases and controls, respectively. A large proportion of cases had a GRS and a MRS in the highest quartile (37.1% and 68.5%, respectively).

**Table 1:** Characteristics of the study population at baseline

Characteristics	Cases (*n* = 143)	Controls (*n* = 1460)	*p* value^a^
Age (years)	63.7 (6.2)	61.8 (6.5)	0.0011
Sex			
Male	90 (62.9)	643 (44.0)	< 0.0001
Smoking status^b^			
Never smoker	17 (12.1)	704 (49.7)	
Former smoker	53 (37.9)	473 (33.4)	
Current smoker	70 (50.0)	239 (16.9)	< 0.0001
Heavy smokers^c^	69 (48.3)	205 (14.0)	< 0.0001
Pack-years^d^	34.8 (24.8)	12.1 (17.5)	< 0.0001
GRS^e^			
Q1 (< 24)	20 (14.0)	319 (21.8)	
Q2 (24, 27)	29 (20.3)	379 (26.0)	
Q3 (27, 30)	41 (28.7)	375 (25.7)	
Q4 (≥ 30)	53 (37.1)	387 (26.5)	0.0110
MRS^e^			
Q1 (< − 0.10)	6 (4.2)	365 (25.0)	
Q2 (− 0.10, + 0.15)	11 (7.7)	365 (25.0)	
Q3 (+ 0.15, + 0.53)	28 (19.6)	365 (25.0)	
Q4 (≥ + 0.53)	98 (68.5)	365 (25.0)	< 0.0001

Means (standard deviation) for continuous variables and *n* (%) for categorical variables

*GRS* genetic risk score, *MRS* methylation risk score, *Q* quartile

^a^Fisher’s exact test for categorical variables and Wilcoxon test for continuous variables

^b^Data missing for 3 cases and 44 controls; never smoker was defined as an adult who has never smoked, or who has smoked less than 100 cigarettes in his or her lifetime; former smoker was defined as an adult who has smoked at least 100 cigarettes in his or her lifetime but who had quit smoking at the time of interview; current smoker was defined as an adult who has smoked 100 cigarettes in his or her lifetime and who currently smokes cigarettes

^c^Heavy smokers were defined as participants with ≥ 30 pack-years of smoking who were either current smokers or had quit smoking ≤ 15 years ago

^d^Data missing for 15 cases and 137 controls

^e^Classified according to quartiles of GRS (MRS) among controls

### Individual associations of GRS and MRS with LC incidence

Table 2 shows the individual associations of GRS and MRS with LC incidence in the entire study population and in the subpopulations including ever or heavy smokers only. Having a score in the top quartile of either score was associated with a significantly increased risk of LC, but associations were much stronger for the MRS. In the analyses among all participants, odds ratios (ORs) (95% confidence interval (CI)) for participants in the top quartile compared to the lowest quartile were 2.18 (1.30–3.81) for the GRS and 20.00 (8.92–53.79) for the MRS in model 1. Additional adjusting for age and sex only in model 2 or for smoking status and pack-years in model 3 reduced the corresponding ORs for MRS to some extent. Nevertheless, with an OR (95% CI) of 5.64 (2.13–17.03), the MRS remained a much stronger and highly significant predictor even after full adjustment for smoking status and pack-years.

**Table 2:** Individual associations of GRS and MRS with LC incidence

Group	Risk score	Quartile^a^	Cases	Controls	OR (95% CI)		
					Model 1^b^	Model 2^c^	Model 3^d^
**All participants**	**GRS**	Q1 (< 24)	20	319	Ref.	Ref.	Ref.
(*N*_case/control_ = 143/1460)		Q2 (24, 27)	29	379	1.22 (0.68–2.23)	1.23 (0.69–2.26)	1.28 (0.66–2.57)
		Q3 (27, 30)	41	375	1.74 (1.01–3.09)	1.60 (0.93–2.86)	1.62 (0.87–3.15)
		Q4 (≥ 30)	53	387	2.18 (1.30–3.81)	2.08 (1.23–3.64)	1.93 (1.05–3.71)
		*p* trend			0.0010	0.0030	0.0251
	**MRS**	Q1 (< − 0.10)	6	365	Ref.	Ref.	Ref.
		Q2 (− 0.10, + 0.15)	11	365	1.91 (0.70–5.75)	1.84 (0.67–5.55)	1.31 (0.43–4.18)
		Q3 (+ 0.15, + 0.53)	28	365	4.96 (2.09–13.79)	4.49 (1.87–12.57)	2.85 (1.09–8.48)
		Q4 (≥ + 0.53)	98	365	20.00 (8.92–53.79)	18.40 (8.08–50.00)	5.64 (2.13–17.03)
		*p* trend			< 0.0001	< 0.0001	< 0.0001
**Ever smokers**	**GRS**	Q1 (< 24)	16	155	Ref.	Ref.	Ref.
(*N*_case/control_ = 123/712)		Q2 (24, 27)	23	171	1.30 (0.67–2.60)	1.30 (0.66–2.61)	1.19 (0.56–2.61)
		Q3 (27, 30)	36	194	1.80 (0.98–3.44)	1.70 (0.92–3.27)	1.70 (0.85–3.58)
		Q4 (≥ 30)	48	192	2.42 (1.35–4.55)	2.43 (1.35–4.60)	2.23 (1.13–4.63)
		*p* trend			0.0013	0.0016	0.0086
	**MRS**	Q1 (< + 0.12)	9	178	Ref.	Ref.	Ref.
		Q2 (+ 0.12, + 0.46)	18	178	2.17 (0.90–5.53)	1.97 (0.81–5.08)	1.75 (0.64–5.13)
		Q3 (+ 0.46, + 0.89)	33	178	4.80 (2.13–11.79)	4.32 (1.89–10.72)	2.48 (0.92–7.21)
		Q4 (≥ + 0.89)	63	178	9.77 (4.49–23.53)	9.58 (4.37–23.28)	3.91 (1.46–11.40)
		*p* trend			< 0.0001	< 0.0001	0.0051
**Heavy smokers**	**GRS**	Q1 (< 24)	8	32	Ref.	Ref.	Ref.
(*N*_case/control_ = 69/205)		Q2 (24, 27)	12	57	0.84 (0.31–2.35)	0.73 (0.26–2.07)	0.75 (0.26–2.19)
		Q3 (27, 30)	24	59	1.63 (0.68–4.24)	1.25 (0.50–3.35)	1.52 (0.59–4.16)
		Q4 (≥ 30)	25	57	1.75 (0.73–4.57)	1.60 (0.65–4.24)	1.62 (0.64–4.38)
		*p* trend			0.0642	0.0982	0.0998
	**MRS**	Q1 (< + 0.47)	6	51	Ref.	Ref.	Ref.
		Q2 (+ 0.47, + 0.86)	15	51	3.25 (1.01–11.66)	3.19 (0.96–11.92)	2.38 (0.67–9.30)
		Q3 (+ 0.86, + 1.32)	20	51	3.31 (1.06–11.51)	3.80 (1.18–13.70)	2.85 (0.84–10.70)
		Q4 (≥ + 1.32)	28	51	4.70 (1.55–16.24)	6.07 (1.92–21.96)	4.26 (1.22–16.52)
		*p* trend			0.0158	0.0042	0.0324

*Q* quartile, *GRS* genetic risk score, *MRS* methylation risk score, *LC* lung cancer, *OR* odds ratio, *CI* confidence interval, *Ref*. reference category

^a^Quartiles of risk score among controls

^b^Model 1: without adjustment for any covariates for GRS and adjusted for batch (3 subsets) and leukocyte composition for MRS

^c^Model 2: like model 1, additionally adjusted for age and sex

^d^Model 3: like model 2, additionally adjusted for smoking status and pack-years

Analyses among ever smokers and heavy smokers showed similar results as those for all participants, although associations for the MRS were somewhat less pronounced compared to those in the entire study population, and associations for the GRS did no longer reach statistical significance in the restricted sample of heavy smokers. In all models and all study populations, the MRS showed substantially stronger associations with LC risk than the GRS.

### Joint associations of GRS and MRS with LC incidence

Table 3 shows the joint associations of GRS and MRS with LC incidence in the entire study population and in the subpopulations (ever or heavy smokers). The joint risk categories were based on two risk groups of GRS and MRS each (low/high risk defined by the median GRS/MRS). The low MRS and low GRS group was assigned as the reference category. In the analyses among all participants, OR (95% CI) for participants in the joint highest group compared to the reference group was 9.08 (4.40–21.35) in model 1. Additional adjusting for age and sex only in model 2 slightly reduced the corresponding OR to some extent. After full adjustment for smoking status and pack-years in model 3, the corresponding OR was 3.50 (1.44–9.50).

**Table 3:** Joint associations of GRS and MRS with LC incidence

Group	MRS risk group^a^	GaRS risk group^a^	Cases	Controls	OR (95%CI)		
					Model 1^b^	Model 2^c^	Model 3^d^
**All participants**	Low	Low	8	368	Ref.	Ref.	Ref.
(*N*_case/control_ = 143/1460)		High	9	362	0.91 (0.33–2.51)	0.86 (0.31–2.38)	0.77 (0.25–2.40)
	High	Low	41	330	5.61 (2.62–13.51)	5.11 (2.36–12.37)	2.29 (0.92–6.32)
		High	85	400	9.08 (4.40–21.35)	7.88 (3.75–18.73)	3.50 (1.44–9.50)
	*p* trend				< 0.0001	< 0.0001	0.0005
**Ever smokers**	Low	Low	10	164	Ref.	Ref.	Ref.
(*N*_case/control_ = 123/712)		High	17	192	1.48 (0.62–3.67)	1.47 (0.61–3.70)	1.38 (0.51–3.95)
	High	Low	29	162	3.99 (1.78–9.61)	4.06 (1.80–9.87)	1.83 (0.68–5.25)
		High	67	194	7.19 ( 3.39-16.69	7.03 (3.26-16.57)	3.35 (1.32-9.30)
	*p* trend				< 0.0001	< 0.0001	0.0038
**Heavy smokers**	Low	Low	6	46	Ref.	Ref.	Ref.
(*N*_case/control_ = 69/205)		High	15	56	2.48 (0.76–9.01)	2.12 (0.62–8.02)	1.78 (0.50–6.89)
	High	Low	14	43	2.81 (0.82–10.77)	3.13 (0.88–12.52)	1.96 (0.53–8.03)
		High	34	60	4.01 (1.33–13.92)	4.44 (1.42–16.06)	3.58 (1.10–13.25)
	*p* trend				0.0225	0.0097	0.0319

*GRS* genetic risk score, *MRS* methylation risk score, *LC* lung cancer, *OR* odds ratio, *CI* confidence interval, *Ref*. reference category

^a^The GRS/MRS median value was used as cut-point for low-/high-risk groups

^b^Model 1: adjusted for batch (3 subsets) and leukocyte composition

^c^Model 2: like model 1, additionally adjusted for age and sex

^d^Model 3: like model 2, additionally adjusted for smoking status and pack-years

Subgroup analyses for ever smokers showed similar results as those for all participants. For heavy smokers, associations were somewhat less distinct compared to those in the entire study population in models 1 and 2. However, similar risk estimates were observed in heavy smokers as those for all participants and ever smokers after full adjustment for smoking status and pack-years in model 3. Within each MRS group, risk of LC was generally higher for participants with high GRS, compared to low GRS, and the highest risk was seen in the joint highest risk group (high MRS and high GRS) in all study populations and models.

### Individual and joint predictive performance of pack-years, GRS, and MRS for LC risk

**Fig. 1:**
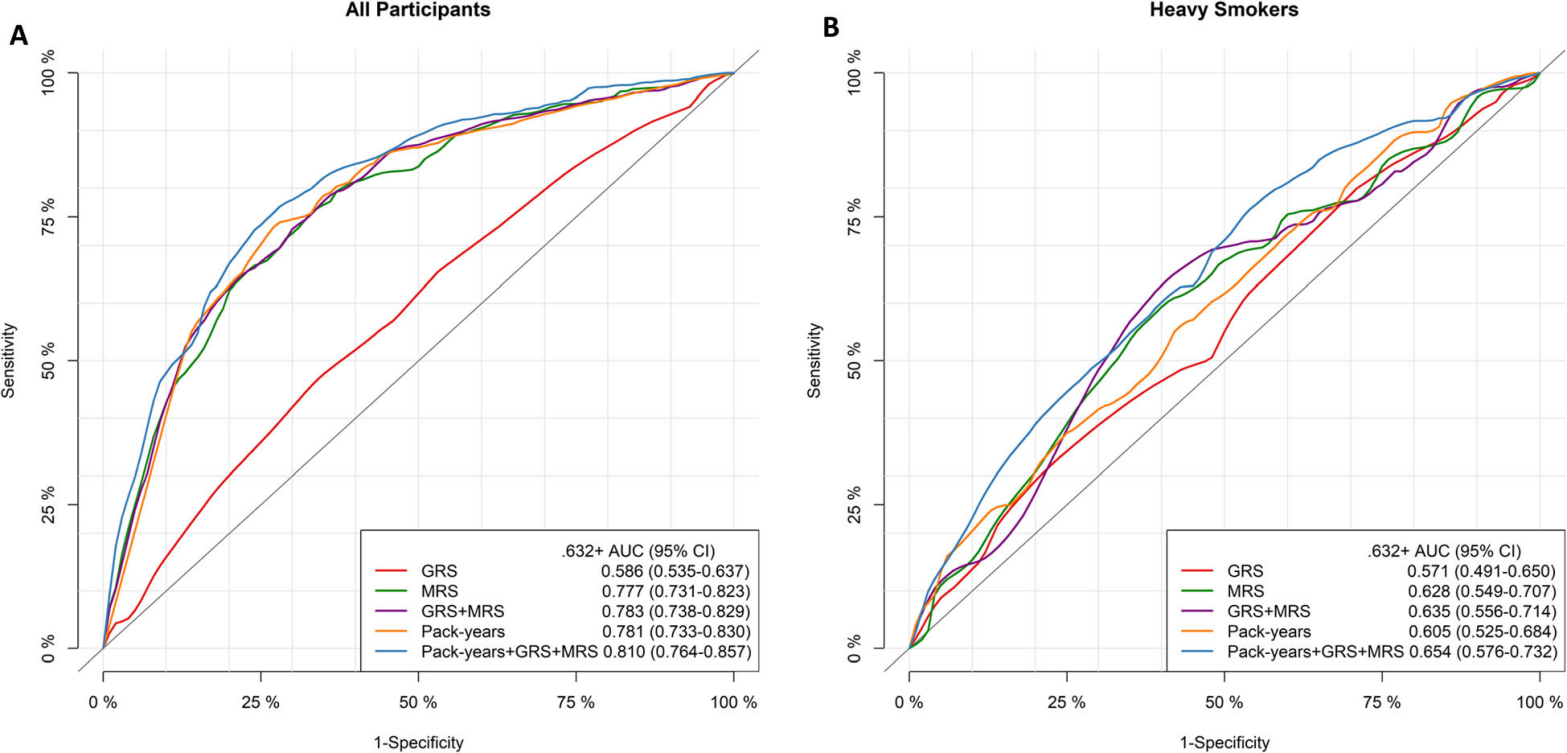
ROC curves for GRS, MRS, pack-years, and their combination in prediction of LC incidence. **a** ROC curves for all participants. **b** ROC curves for heavy smokers

Table 4 and Fig. 1 display the individual and joint predictive performance of pack-years, GRS, and MRS for LC risk. In the entire study population as well as within the subgroups of ever and heavy smokers, AUCs were generally the lowest for GRS alone, whereas pack-years, MRS, and combination of MRS and GRS generally provided similar predictive performance. Prediction performance was substantially improved when adding MRS to models based on pack-years alone. Optimism-corrected AUCs for joint inclusion of pack-years and MRS compared to models including pack-years only increased from 0.781 to 0.812, from 0.701 to 0.728, and from 0.605 to 0.652 in the entire study population and within the subpopulations of ever smokers and heavy smokers, respectively. Additional inclusion of GRS led to at best modest further improvement of AUCs. However, compared to models including pack-years only, NRIs for models including pack-years, GRS, and MRS were 13.9% (*p* = 0.02), 16.6% (*p* = 0.02), and 26.7% (*p* = 0.01), and IDIs were 2.6%, 3.0%, and 3.4% (all *p* values < 0.01) among all study participants, ever smokers, and heavy smokers, respectively. Corresponding confusion matrix and precision-recall curves showed consistent performance, where the best predictive performance was achieved by the joint inclusion of pack-years, MRS, and GRS, especially for heavy smokers (Table S3 and Figure S1).

**Table 4:** Individual and joint predictive performance of pack-years, GRS, and MRS for LC risk

Predictor	AUC (95% CI)		NRI^a^		IDI^a^	
	Apparent	.632+	%	*p* value	%	*p* value
**All participants**^b^						
GRS	0.587 (0.536–0.638)	0.586 (0.535–0.637)				
MRS	0.777 (0.731–0.823)	0.777 (0.731–0.823)				
GRS + MRS	0.784 (0.738–0.830)	0.783 (0.738–0.829)				
Pack-years	0.782 (0.733–0.830)	0.781 (0.733–0.830)				
Pack-years + GRS	0.779 (0.730–0.827)	0.777 (0.728–0.826)	7.5	0.0146	0.86	0.0082
Pack-years + MRS	0.813 (0.767–0.859)	0.812 (0.766–0.858)	12.3	0.0355	1.99	0.0157
Pack-years + GRS + MRS	0.813 (0.767–0.859)	0.810 (0.764–0.857)	13.9	0.0198	2.59	0.0012
**Ever smokers**^c^						
GRS	0.593 (0.536–0.649)	0.592 (0.536–0.649)				
MRS	0.696 (0.642–0.751)	0.696 (0.641–0.751)				
GRS + MRS	0.721 (0.667–0.775)	0.717 (0.663–0.770)				
Pack-years	0.702 (0.644–0.759)	0.701 (0.644–0.759)				
Pack-years + GRS	0.708 (0.651–0.765)	0.705 (0.647–0.762)	12.7	0.0097	1.39	0.0059
Pack-years + MRS	0.731 (0.675–0.787)	0.728 (0.672–0.784)	16.6	0.0100	1.89	0.0104
Pack-years + GRS + MRS	0.743 (0.687–0.798)	0.737 (0.681–0.792)	16.6	0.0163	2.96	0.0002
**Heavy smokers**^d^						
GRS	0.574 (0.494–0.654)	0.571 (0.491–0.650)				
MRS	0.629 (0.550–0.707)	0.628 (0.549–0.707)				
GRS + MRS	0.649 (0.570–0.727)	0.635 (0.556–0.714)				
Pack-years	0.605 (0.525–0.684)	0.605 (0.525–0.684)				
Pack-years + GRS	0.619 (0.540–0.698)	0.610 (0.531–0.690)	14.5	0.0598	1.52	0.0456
Pack-years + MRS	0.662 (0.584–0.740)	0.652 (0.574–0.730)	19.4	0.0357	2.13	0.0322
Pack-years + GRS + MRS	0.672 (0.595–0.749)	0.654 (0.576–0.732)	26.7	0.0106	3.35	0.0036

*GRS* genetic risk score, *MRS* methylation risk score, *LC* lung cancer, *AUC* area under the curve, *CI* confidence interval, *NRI* net reclassification improvement, *IDI* integrated discrimination improvement

^a^NRI and IDI were estimated between combined models including pack-years and risk scores and the pack-years only model

^b^Case/control number 143/1460 in all participants

^c^Case/control number 123/712 in ever smokers

^d^Case/control number 69/205 in heavy smokers

## Discussion

We have evaluated the individual and joint potential of a GRS based on 31 SNPs and a MRS based on 151 smoking-associated CpGs for enhancing risk stratification in LC prevention. We found moderate associations of the GRS and strong associations of the MRS with the risk of LC even after comprehensive adjustment for smoking history in a case-control study that was nested in a general population-based cohort study. Furthermore, we demonstrated that the addition of MRS and GRS strongly enhanced the risk prediction compared to the standard risk stratification by pack-years both within the entire study population as well as within the high-risk groups of ever smokers and heavy smokers, the improvement being mostly attributable to the inclusion of the MRS. Our results provide support for including MRS, potentially along with GRS, into LC risk assessment models to more accurately stratify individuals and select those at highest risk for inclusion in screening programs.

LDCT is an effective screening tool for early detection of LC and is recommended by the United States Preventive Services Task Force to screen for LC among high-risk heavy smokers (aged 55–80 years, with ≥ 30 pack-years of smoking who are either current smokers or have quit smoking ≤ 15 years ago) [7]. However, false positive results, high costs, and potential radiation exposure remain major concerns for LDCT-based screening [7, 22]. This underlines the importance of identifying individuals at highest risk to maximize the benefits and minimize the harms of screening. To facilitate this, multiple risk prediction models using traditional factors like age, sex, family history, smoking history, occupational exposure, etc. have been proposed [23–25]. Traditional smoking-based risk models have mostly used self-reported smoking exposure information, which may not accurately represent the actual exposure [26, 27]. Biomarkers, reflecting biologically relevant smoking exposure, such as smoking-associated DNA methylation markers, might therefore be most useful to improve current LC risk stratification based on self-reported smoking history. In addition, studies have demonstrated that methylation changes at smoking-associated genes rather than at the LC-related genes were involved in the initiation of LC [18, 19, 28]. Therefore, smoking-associated CpGs might be more suitable for LC screening than LC-related CpGs. Unlike DNA methylation, SNPs do not vary over time and are not affected by exposures and disease status [29]. The SNPs identified by GWAS may be associated with either the initiation or progression of LC (or both), regardless of their location at smoking-associated genes or elsewhere. By incorporating all LC-associated SNPs into GRS and smoking-associated CpGs into MRS, this study comprehensively evaluated the genetic and epigenetic effects in LC risk stratification.

During the last decade, large-scale GWAS [9, 30, 31] have identified numerous LC susceptibility loci. Prior studies have explored the value of genetic variants in LC risk prediction [32–34]. Cheng et al. [34] developed a risk model that included both a risk score based on 38 genetic variants (selected from 241 genetic variants identified in large-scale studies of ethnically diverse populations) and self-reported smoking information, which was evaluated in a training and testing set. In the testing set, a modest improvement in AUC for a model that included both a GRS and smoking information (AUC = 0.647), compared with a model that included smoking information only (AUC = 0.625), was reported. In a study by Qian et al. [33], inclusion of 301 GWAS detected SNPs barely improved prediction performance of a model that included epidemiologic factors (age, sex, and pack-years) only (AUC 0.617 vs. 0.607 in the test set). In our study, adding a GRS based on 31 GWAS-identified SNPs to a model based on pack-years likewise yielded at best only a very limited increase in AUCs. However, relevant increases were achieved by including the MRS, with models adding either MRS alone or both GRS and MRS yielding substantial discrimination improvement as indicated by NRI and IDI.

Over the past several years, a number of studies have highlighted the value of smoking-associated DNA methylation biomarkers assessed in blood for LC prediction [21, 35–38]. Few studies have investigated the degree of improvement of prediction models based on smoking-associated DNA methylation markers beyond self-reported smoking exposure. Baglietto et al. [38] estimated the gain in prediction accuracy of a model additionally including 6 smoking-associated CpGs to a model only including self-reported smoking information (smoking status, pack-years) in two cohorts and reported increases in AUCs of 0.026 and 0.055, respectively, in the discovery studies from which the MRS was derived. In previous analyses of the ESTHER study which had focused on one or three DNA methylation markers only, substantially lower improvements of prediction accuracy had been observed [21, 36]. The results of the current study suggest that using more comprehensive MRS may substantially improve risk prediction.

Our current study differs from prior studies by evaluating not only the predictive value of genetic or methylation information individually but also by comparing their predictive value and evaluating their combined predictive value for LC prediction beyond prediction by pack-years alone. While GRS showed modest predictive value for LC risk, the predictive value of MRS was much higher, and addition of GRS to models including MRS did not improve the predictive value in the entire study population. Nevertheless, among the risk groups of ever and heavy smokers, the combination of pack-years with both GRS and MRS resulted in the highest predictive performance.

Our results may have important clinical implications for LC screening and preventive strategies. Our results suggested that epigenetic signatures may have the potential to better select patients at highest risk for LC screening. Future research should explore possibilities to further enhance prediction by more refined MRS and possible combinations with other potentially promising epigenetic signatures, such as microRNA signatures [39, 40]. Future studies should also address the acceptance, feasibility, and cost-effectiveness of such risk stratification in LC screening programs.

To our knowledge, this is the first study to comprehensively evaluate the individual and joint performance of GRS and MRS for predicting LC risk in a prospective cohort with 14-year follow-up. Additionally, detailed information on smoking was available which enabled evaluation of the predictive value beyond the currently recommended, exclusively smoking-based criteria for selecting people for LC screening. However, our study also has some important limitations. In particular, despite the large size of the cohort, this study was based on the limited number of LC cases hindered more detailed analyses by important factors, such as age and sex of the study population, time between blood sampling and LC occurrence, or different LC subtypes. Potential variation of predictive performance by such factors should be evaluated in even larger studies. Furthermore, although our estimates of prediction performance of combinations of predefined MRS, GRS, and smoking history were internally corrected for potential over-optimism by bootstrapping, further validation in independent cohorts is warranted. Future, ideally much larger studies should also address the performance of GRS and MRS in predicting risk of specific genetic variants of lung cancer.

## Conclusion

In summary, despite its limitations, this study provides evidence for the potential of GRS and particularly MRS, by themselves and in combination, for enhancing LC risk stratification. To our knowledge, this is the first prospective cohort study evaluating both types of scores in direct comparison and combination. We showed that, although both GRS and MRS predicted LC risk, predictive value, especially predictive value beyond smoking history was much stronger for MRS than for GRS. The predictive value of MRS based on a large number of established smoking-related CpGs investigated in this study also outperformed the previously demonstrated predictive value of a few single CpGs. Consideration of MRS, by itself or in combination with GRS, may therefore have the potential to enhance risk stratification for LC screening. Further research is warranted to replicate and expand our results in larger and ethnically diverse populations and include screening cohorts in order to more comprehensively evaluate the potential of the risk scores for identifying high-risk individuals for LC screening. Future studies should also aim for identification of additional genetic or epigenetic markers and integration of additional environmental or life-style factors into the risk-prediction models in order to further enhance risk stratification for LC and pave the way for better targeting LC screening offers to those at highest risk.

## Methods

### Study population and data collection

**Fig. 2:**
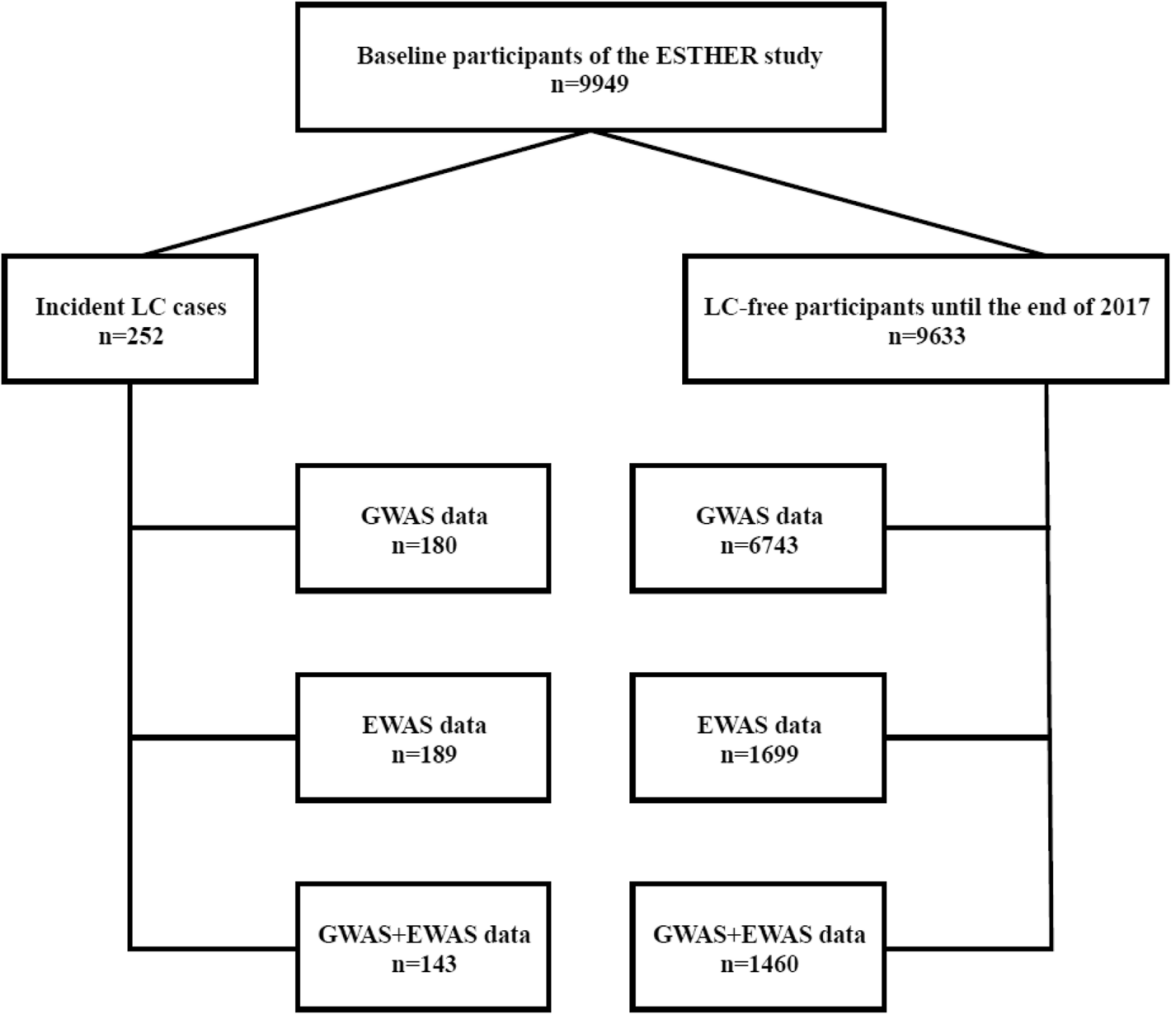
Flow diagram of study participants

We selected study subjects from the ESTHER study, an ongoing population-based cohort study conducted in Saarland, Germany. Details of the ESTHER study design have been described previously [41]. Briefly, 9949 participants aged 50–75 years were recruited between July 2000 and December 2002 by their general practitioners in the context of a general health screening examination, and they have been regularly followed-up thereafter. Information on socio-demographic characteristics, lifestyle factors, and health status at baseline was obtained by standardized self-administered questionnaires. Detailed smoking history was obtained at recruitment, including smoking status, years of initiation and cessation (if applicable), and average number of cigarettes smoked per day. In addition, biological samples (blood, stool, and urine) were collected and stored at – 80 °C until analysis. Prevalent and incident cancer was determined by record linkage with data from the Saarland Cancer Registry. The study was approved by the ethics committees of the University of Heidelberg and of the state medical board of Saarland, Germany. All participants provided written informed consent. The analysis is based on a case-control study nested within the ESTHER cohort using data and biospecimen collected at baseline. We included 143 participants with incident LC (ICD10: C34) and 1460 participants without diagnosis of LC until the end of 2017, for whom both GWAS and EWAS data was available (Fig. 2).

### Genotyping

DNA was extracted from whole blood samples using a salting out procedure [42] and genotyped using the Illumina Infinium OncoArray BeadChip (Illumina, San Diego, CA). General genotyping quality control assessment was performed as previously described [43]. Genotypes for common variants were imputed using as reference Dataset the 1000 Genomes Project (GP) (phase 3, Oct. 2014) for chromosomes 1 to 22 with IMPUTE v2.3.2 after pre-phasing with SHAPEIT v2.12. Thresholds were set for imputation quality to retain both common and rare variants for validation. In detail, poorly imputed SNPs defined by an information metric I < 0.70 were excluded for the subsequent analysis. All genomic locations are given in GRCh37 (hg19) coordinates. All SNPs having a minor allele frequency (MAF) < 1% were excluded. After imputation, the SNP set consisted of 9,198,808 successfully genotyped and imputed SNPs. PLINK v1.90b6.9 was then used to extract SNPs for the required regions of interest [44].

### Methylation assessment

Methylation of DNA extracted from whole blood was quantified using the Infinium HumanMethylation 450K BeadChip Assay (Illumina, San Diego, CA) as previously described [28]. Briefly, 1.5 μg DNA was bisulfite converted, and 200 ng bisulfite-treated DNA was applied with the 450K BeadChips following the manufacturer’s instruction. Illumina’s GenomeStudio® (version 2011.1; Illumina Inc.) was used to extract DNA methylation signals from the scanned arrays (module version 1.9.0; Illumina Inc.) and to calculate methylation β-values. Data were normalized to internal controls provided by the manufacturer. In addition, probes with detection *p* values > 0.05, with missing values > 10%, or targeting the sex chromosomes were excluded from analysis.

### Statistical analysis

#### Genetic risk score (GRS)

We built a GRS using a set of 51 SNPs from published GWAS on LC in European populations, summarized by Bosse et al. [9] (Table S1). SNPs were excluded from further analyses if their imputation resulted in missing values in > 10% of our samples, or with a MAF < 0.5% in European populations. If SNPs were in high linkage disequilibrium (LD; D′ ≥ 0.95 and *r*^2^ ≥ 0.80) with each other in 200 kb, we only selected the most significant one in our sample for GRS building. Twenty SNPs were hence ruled out, which left 31 SNPs for the further calculation. The GRS values for each participant were calculated as the sum of the risk alleles across the 31 SNPs.

#### Methylation risk score (MRS)

A set of 151 smoking-associated CpGs that had been identified ≥ 2 times in previous smoking EWAS [20] was used to build the MRS (Table S2). The MRS for each participant was calculated according to an algorithm proposed by Teschendorff et al. [45]:

$$ \mathrm{MRS}=\frac{1}{n}{\sum}_c^n{W}_c\frac{\beta_{cs}-{\mu}_c}{\sigma_c}, $$

where *n* is the number of CpGs included in score calculation, i.e. 151, and μ_c_ and σ_c_ are the mean methylation β-value and the standard deviation of each of the 151 CpGs among never smokers (the reference), respectively. *β*_cs_ is the methylation β-value of each CpG site, *c*, for each participant. *W*_c_ is + 1 (− 1) if the CpG, *c*, is hypermethylated (hypomethylated) in smokers. In subsequent analyses, *W*_c_ was derived from Joehanes et al.’s study (Table S2) [46].

#### Associations of GRS and MRS with LC risk

The associations of the risk scores with LC incidence were assessed by logistic regression models, first without adjusting for any confounders for GRS and adjusting for batch (3 subsets) and leukocyte composition only [47] for MRS (model 1), additionally adjusting for age and sex only (model 2), and further additionally adjusting for smoking status (never/former smoker, current smoker) and lifetime cumulative smoking intensity (pack-years) (model 3). GRS or MRS was included in the models as categorical variables (participants classified according to quartiles of risk score among controls). In order to assess the potential of risk prediction within the risk group of ever smokers or within the high-risk group of heavy smokers who are commonly recommended to undergo LC screening [7], associations of risk scores with incident LC were furthermore examined separately among ever smokers (current or former smokers) and heavy smokers (participants with ≥ 30 pack-years of smoking who were either current smokers or ever smokers who had quit smoking ≤ 15 years ago [7]).

#### Predictive performance of pack-years, GRS, and MRS for LC incidence

The individual and joint performance of pack-years, GRS, and MRS in predicting LC incidence was assessed by areas under the receiver operating characteristic curve (AUCs). In these analyses, GRS and MRS were entered as quantitative variables. Potential over-optimism was corrected by the 0.632+ bootstrapping method [48] with 1000 replications using the R package “ModelGood.” To evaluate the predicted probability changes for subjects in the correct direction between two models, continuous net reclassification improvement (NRI) [49] with threshold of probability changes by at least 5% was estimated using the R package “nricens.” To assess how the discriminating ability improved between two models, integrated discrimination improvement (IDI) [50] was estimated using the R package “PredictABEL.”

All statistical analyses were conducted by the R software, version 3.5.3 (R Foundation, Vienna, Austria). Two-sided *p* values of < 0.05 were considered statistically significant.

## Supplementary information

**Additional file 1: Table S1.** SNPs used to construct the genetic risk score. **Table S2.** Smoking-associated CpGs used to construct the methylation risk score. **Table S3.** Confusion matrix for GRS, MRS, pack-years and their combination in LC risk prediction. **Figure S1.** Precision-recall curves for GRS, MRS, pack-years and their combination in prediction of LC incidence.

## Data Availability

Anonymized data used for this study are available from the corresponding author upon reasonable request.

## Abbreviations

AUC: Area under the receiver operating characteristic curve
CpG: Cytosine-phosphate-guanine
EWAS: Epigenome-wide association study
GWAS: Genome-wide association study
GRS: Genetic risk score
IDI: Integrated discrimination improvement
LC: Lung cancer
MRS: Methylation risk score
NRI: Net reclassification improvement
SNP: Single-nucleotide polymorphism
