# Supplementary material for: Individual and joint contributions of genetic and methylation risk scores for enhancing lung cancer risk stratification: data from a population-based cohort in Germany
Source: Clin Epigenetics. 2020 Jun 18;12:89. doi: 10.1186/s13148-020-00872-y (PMC7301507; doi:10.1186/s13148-020-00872-y)
Supplement: Supplementary file 1 — Additional file 1: Table S1. SNPs used to construct the genetic risk score. Table S2. Smoking-associated CpGs used to construct the methylation risk score. Table S3. Confusion matrix for GRS, MRS, pack-years and their combination in LC risk prediction. Figure S1. Precision-recall curves for GRS, MRS, pack-years and their combination in prediction of LC incidence. [file 13148_2020_872_MOESM1_ESM.docx]

Supplementary Appendix to

**Individual and Joint Contributions of Genetic and Methylation Risk Scores**

**for Enhancing Lung Cancer Risk Stratification: data from a population-based cohort in Germany**

Haixin Yu^1,2^, Janhavi R. Raut^2,3^, Ben Schöttker^1,4^, Bernd Holleczek^5^, Yan Zhang^1,6^, Hermann Brenner^1,3,6^

^1^Division of Clinical Epidemiology and Aging Research, German Cancer Research Center (DKFZ), Im Neuenheimer Feld 581, 69120 Heidelberg, Germany

^2^Medical Faculty Heidelberg, University of Heidelberg, Im Neuenheimer Feld 672, 69120 Heidelberg, Germany

^3^Division of Preventive Oncology, German Cancer Research Center (DKFZ) and National Center for Tumor Diseases (NCT), Im Neuenheimer Feld 460, 69120 Heidelberg, Germany

^4^Network Aging Research, University of Heidelberg, Bergheimer Straße 20, 69115 Heidelberg, Germany

^5^Saarland Cancer Registry, Krebsregister Saarland, Präsident-Baltz-Straße 5, 66119 Saarbrücken, Germany

^6^German Cancer Consortium (DKTK), German Cancer Research Center (DKFZ), Im Neuenheimer Feld 280, 69120 Heidelberg, Germany

**Table of Contents**

**Table S1, pages 3-5**

**Table S2, pages 6-9**

**Table S3, page 10**

**Figure S1, page 11**

**References, pages 12-13**

| **Table S1.** SNPs used to construct the genetic risk score (summarized by Bosse et al. [1]) | | | | | | | | |  |
| --- | --- | --- | --- | --- | --- | --- | --- | --- | --- |
| **#** | **Region** | **SNP** | **Position (hg19)** | **Gene** | **Risk / other allele** | **OR for minor allele in discovery study** | **MAF in European populations** | **Discovered by** | **Validated by** |
| 1 | 1p31.1 | rs71658797 | 1:77967507 | *AK5* | A / T | 1.14 | A=0.086 | [2] |  |
| 2 | 2q32.1 | rs11683501 | 2:184159036 | */* | G / A | 1.17 | G=0.486 | [3] |  |
| 3 | 3q28 | rs13080835 | 3:189357199 | *TP63* | G / T | 1.11 | T=0.494 | [2] |  |
| 4 | 3q28 | rs13314271* | 3:189357602 | *TP63* | C / T | 1.06 | C=0.494 | [4] |  |
| 5 | 3q28 | rs4488809* | 3:189356261 | *TP63* | C / T | 1.06 | C=0.494 | [4] |  |
| 6 | 5p15 | rs2736100 | 5:1286516 | *TERT* | C / A | 1.17 | C=0.499 | [5] | [3, 6] |
| 7 | 5p15 | rs401681* | 5:1322087 | *CLPTM1L* | G / A | 1.14 | A=0.441 | [7] | [3] |
| 8 | 5p15.33 | rs2853677 | 5:1287194 | *TERT* | G / A | 1.33 | G=0.413 | [3] |  |
| 9 | 5p15 | rs31489 | 5:1342714 | *CLPTM1L* | C / A | 1.12 | A=0.408 | [6] |  |
| 10 | 5p15.33 | rs402710* | 5:1320722 | *CLPTM1L* | T / C | 1.21 | T=0.332 | [5] |  |
| 11 | 5p15 | rs4635969 | 5:1308552 | */* | G / A | 1.15 | A=0.188 | [6] |  |
| 12 | 5p15.33 | rs465498* | 5:1325803 | *CLPTM1L* | A / G | 1.15 | G=0.441 | [3] |  |
| 13 | 5p15.33 | rs4975616 | 5:1315660 | */* | A / G | 1.15 | G=0.424 | [8] |  |
| 14 | 5p15.33 | rs7705526* | 5:1285974 | *TERT* | A / C | 1.25 | A=0.337 | [2] |  |
| 15 | 6p21.33 | rs3117582* | 6:31620520 | *APOM, BAG6* | C / A | 1.30 | G=0.075 | [7] | [3, 6, 8] |
| 16 | 6p21.33 | rs1150752 | 6:32064726 | *TNXB* | C / T | 1.24 | C=0.075 | [8] |  |
| 17 | 6p21.33 | rs116822326* | 6:31434111 | */* | G / A | 1.25 | G=0.140 | [2] |  |
| 18 | 6p21-22 | rs2523546* | 6:31332920 | */* | A / G | 1.32 | A=0.087 | [3] |  |
| 19 | 6p21-22 | rs2523571 | 6:31329691 | */* | A / T | 1.32 | A=0.084 | [3] |  |
| 20 | 6p21.33 | rs3131379* | 6:31721033 | *MSH5-SAPCD1, MSH5* | A / G | 1.26 | A=0.075 | [7] |  |
| 21 | 6q27 | rs6920364 | 6:167376466 | */* | C / G | 1.07 | C=0.496 | [2] |  |
| **Table S1.** Continued | | | | | | | | | |
| 22 | 8p21.1 | rs11780471 | 8:27344719 | */* | G / A | 1.15 | A=0.075 | [2] |  |
| 23 | 8p12 | rs4236709 | 8:32410110 | *NRG1* | G / A | 1.13 | G=0.189 | [2] |  |
| 24 | 9p21.3 | rs1333040 | 9:22083404 | *CDKN2B-AS1* | C / T | 1.06 | C=0.426 | [3] |  |
| 25 | 9p21.3 | rs1537372 | 9:22103183 | *CDKN2B-AS1* | G / T | 1.14 | T=0.438 | [3] |  |
| 26 | 9p21.3 | rs885518 | 9:21830157 | *MTAP* | G / A | 1.17 | G=0.111 | [2] |  |
| 27 | 10q24.3 | rs11591710 | 10:105687632 | */* | C / A | 1.16 | C=0.125 | [2] |  |
| 28 | 11q23.3 | rs1056562 | 11:118125625 | *MPZL2* | T / C | 1.11 | T=0.480 | [2] |  |
| 29 | 12p13.33 | rs10849605 | 12:1064438 | *RAD52* | C / T | 1.09 | T=0.491 | [3] |  |
| 30 | 12p13.33 | rs3748522* | 12:1058688 | *RAD52* | C / A | 1.16 | A=0.481 | [3] |  |
| 31 | 12p13.33 | rs7953330 | 12:998819 | *WNK1* | G / C | 1.16 | C=0.293 | [2] |  |
| 32 | 13q13.1 | rs11571833 | 13:32972626 | *BRCA2* | T / A | 1.83 | T=0.011 | [4] | [2] |
| 33 | 13q13.1 | rs56084662 | 13:32869864 | *FRY* | A / G | 2.01 | A=0.007 | [4] |  |
| 34 | 15q25 | rs1051730* | 15:78894339 | *CHRNA3* | A / G | 1.32 | A=0.369 | [9-11] | [3, 5, 6, 12] |
| 35 | 15q25 | rs8034191* | 15:78806023 | *HYKK* | C / T | 1.32 | C=0.375 | [9, 11] | [3, 6, 8, 12] |
| 36 | 15q25.1 | rs12914385 | 15:78898723 | *CHRNA3* | T / C | 1.29 | T=0.405 | [8] | [6] |
| 37 | 15q25 | rs16969968* | 15:78882925 | *CHRNA5* | A / G | 1.30 | A=0.366 | [9] | [12] |
| 38 | 15q25.1 | rs8042374 | 15:78908032 | *CHRNA3* | A / G | 1.33 | G=0.241 | [7] | [8] |
| 39 | 15q25.1 | rs55781567 | 15:78857986 | *CHRNA5* | G / C | 1.30 | G=0.376 | [2] |  |
| 40 | 15q24-25.1 | rs578776* | 15:78888400 | *CHRNA3* | G / A | 1.27 | A=0.281 | [12] |  |
| 41 | 15q25 | rs6495306* | 15:78865893 | *CHRNA5* | A / G | 1.10 | G=0.388 | [3] |  |
| 42 | 15q25 | rs6495309* | 15:78915245 | *CHRNA3, CHRNB4* | C / T | 1.25 | T=0.231 | [3] |  |
| 43 | 15q21.1 | rs66759488 | 15:47577451 | *SEMA6D* | A / G | 1.07 | A=0.357 | [2] |  |
| 44 | 15q25 | rs680244 | 15:78871288 | *CHRNA5* | C / T | 1.11 | T=0.390 | [3] |  |
| 45 | 15q21.1 | rs77468143 | 15:49376624 | */* | T / G | 1.16 | G=0.280 | [2] |  |
| 46 | 15q21.1 | rs931794* | 15:78826180 | *HYKK* | G / A | 1.13 | G=0.383 | [11] |  |
| **Table S1.** Continued | | | | | | | | | |
| 47 | 15q21.1 | rs938682* | 15:78896547 | *CHRNA3* | A / G | 1.33 | G=0.242 | [8] |  |
| 48 | 15q25 | rs951266* | 15:78878541 | *CHRNA5* | A / G | 1.31 | A=0.366 | [3] |  |
| 49 | 19q13.2 | rs56113850 | 19:41353107 | *CYP2A6* | C / T | 1.14 | T=0.408 | [2] |  |
| 50 | 20q13.33 | rs41309931 | 20:62326579 | *RTEL1-TNFRSF6B, RTEL1, TNFRSF6B* | T / G | 1.17 | T=0.105 | [2] |  |
| 51 | 22q12.1 | rs17879961 | 22:29121087 | *CHEK2* | A / G | 1.67 | G=0.005 | [4] | [2] |

* SNPs not included in the GRS due to linkage disequilibrium (D'≥0.95 and r2≥0.80) with another SNP. ** stated by Phase 3 (Version 5) of the 1000 Genomes Project.

Abbreviations: LC, lung cancer; SNP, single-nucleotide polymorphism; OR, odds ratio; MAF, minor allele frequency.

| **Table S2.** Smoking-associated CpGs used to construct the methylation risk score (summarized by Gao et al. [13]) | | | | |
| --- | --- | --- | --- | --- |
| **#** | **CpGs** | **Position (hg19)** | **Gene** | **Weight*** |
| 1 | cg04885881 | chr01:11,123,118 | *Unknown* | -1 |
| 2 | cg21393163 | chr01:12,217,630 | *Unknown* | -1 |
| 3 | cg09069072 | chr01:15,482,754 | *TMEM51* | -1 |
| 4 | cg21913886 | chr01:15,485,346 | *TMEM51* | -1 |
| 5 | cg11231349 | chr01:162,050,657 | *NOS1AP* | -1 |
| 6 | cg19713429 | chr01:19,810,690 | *CAPZB* | -1 |
| 7 | cg09469355 | chr01:2,161,887 | *SKI* | -1 |
| 8 | cg08709672 | chr01:206,224,335 | *AVPR1B* | -1 |
| 9 | cg20295214 | chr01:206,226,795 | *AVPR1B* | -1 |
| 10 | cg03547355 | chr01:227,003,061 | *Unknown* | -1 |
| 11 | cg11314684 | chr01:244,006,289 | *AKT3* | -1 |
| 12 | cg27537125 | chr01:25,349,681 | *Unknown* | -1 |
| 13 | cg21140898 | chr01:51,442,318 | *Unknown* | -1 |
| 14 | cg25189904 | chr01:68,299,493 | *GNG12* | -1 |
| 15 | cg26764244 | chr01:68,299,511 | *GNG12* | -1 |
| 16 | cg12547807 | chr01:9,473,751 | *Unknown* | -1 |
| 17 | cg10399789 | chr01:92,945,668 | *GFI1* | -1 |
| 18 | cg09662411 | chr01:92,946,132 | *GFI1* | -1 |
| 19 | cg18146737 | chr01:92,946,701 | *GFI1* | -1 |
| 20 | cg12876356 | chr01:92,946,825 | *GFI1* | -1 |
| 21 | cg18316974 | chr01:92,947,035 | *GFI1* | -1 |
| 22 | cg09935388 | chr01:92,947,588 | *GFI1* | -1 |
| 23 | cg26271591 | chr02:178,125,956 | *NFE2L2* | -1 |
| 24 | cg23667432 | chr02:233,244,439 | *ALPP* | -1 |
| 25 | cg19713851 | chr02:233,246,594 | *ALPP* | -1 |
| 26 | cg27241845 | chr02:233,250,371 | *Unknown* | -1 |
| 27 | cg03329539 | chr02:233,283,329 | *Unknown* | -1 |
| 28 | cg06644428 | chr02:233,284,113 | *Unknown* | -1 |
| 29 | cg05951221 | chr02:233,284,402 | *Unknown* | -1 |
| 30 | cg21566642 | chr02:233,284,662 | *Unknown* | -1 |
| 31 | cg01940273 | chr02:233,284,935 | *Unknown* | -1 |
| 32 | cg13193840 | chr02:233,285,289 | *Unknown* | -1 |
| 33 | cg26718213 | chr02:241,976,081 | *SNED1* | +1 |
| 34 | cg23079012 | chr02:8,343,711 | *Unknown* | -1 |
| 35 | cg17024919 | chr03:21,792,248 | *ZNF385D* | -1 |
| 36 | cg23480021 | chr03:22,412,746 | *Unknown* | +1 |
| 37 | cg03274391 | chr03:22,413,232 | *Unknown* | +1 |
| 38 | cg00501876 | chr03:39,193,252 | *CSRNP1* | -1 |
| 39 | cg18642234 | chr03:49,394,623 | *GPX1* | -1 |
| 40 | cg18754985 | chr03:98,237,751 | *CLDND1* | -1 |
| 41 | cg19859270 | chr03:98,251,295 | *GPR15* | -1 |
| 42 | cg02657160 | chr03:98,311,063 | *CPOX* | -1 |
| 43 | cg24556382 | chr04:174,173,456 | *GALNT7* | -1 |
| 44 | cg21121843 | chr04:3,203,983 | *HTT* | -1 |
| **Table S2.** Continued | | | | |
| 45 | cg14580211 | chr05:150,161,300 | *C5orf62* | -1 |
| 46 | cg12513616 | chr05:177,370,977 | *Unknown* | -1 |
| 47 | cg06060868 | chr05:231,934 | *SDHA* | -1 |
| 48 | cg13039251 | chr05:32,018,602 | *PDZD2* | +1 |
| 49 | cg11554391 | chr05:321,320 | *AHRR* | -1 |
| 50 | cg17924476 | chr05:323,795 | *AHRR* | +1 |
| 51 | cg12806681 | chr05:368,395 | *AHRR* | -1 |
| 52 | cg03991871 | chr05:368,448 | *AHRR* | -1 |
| 53 | cg23916896 | chr05:368,805 | *AHRR* | -1 |
| 54 | cg11902777 | chr05:368,843 | *AHRR* | -1 |
| 55 | cg01899089 | chr05:369,969 | *AHRR* | -1 |
| 56 | cg23576855 | chr05:373,300 | *AHRR* | -1 |
| 57 | cg05575921 | chr05:373,378 | *AHRR* | -1 |
| 58 | cg26703534 | chr05:377,358 | *AHRR* | -1 |
| 59 | cg01097768 | chr05:378,855 | *AHRR* | -1 |
| 60 | cg14817490 | chr05:392,920 | *AHRR* | -1 |
| 61 | cg17287155 | chr05:393,347 | *AHRR* | -1 |
| 62 | cg04551776 | chr05:393,366 | *AHRR* | -1 |
| 63 | cg25648203 | chr05:395,445 | *AHRR* | -1 |
| 64 | cg21161138 | chr05:399,361 | *AHRR* | -1 |
| 65 | cg03604011 | chr05:400,201 | *AHRR* | +1 |
| 66 | cg24090911 | chr05:400,732 | *AHRR* | -1 |
| 67 | cg05673882 | chr05:74,862,702 | *POLK* | -1 |
| 68 | cg20778199 | chr06:148,020,881 | *Unknown* | -1 |
| 69 | cg00931843 | chr06:155,442,993 | *TIAM2* | +1 |
| 70 | cg06126421 | chr06:30,720,081 | *Unknown* | -1 |
| 71 | cg14753356 | chr06:30,720,109 | *Unknown* | -1 |
| 72 | cg24859433 | chr06:30,720,204 | *Unknown* | -1 |
| 73 | cg15342087 | chr06:30,720,210 | *Unknown* | -1 |
| 74 | cg17619755 | chr06:31,760,629 | *VARS* | +1 |
| 75 | cg15474579 | chr06:36,645,813 | *CDKN1A* | -1 |
| 76 | cg09837977 | chr07:110,731,202 | *LRRN3;IMMP2L* | -1 |
| 77 | cg11556164 | chr07:110,738,316 | *LRRN3;IMMP2L* | -1 |
| 78 | cg05221370 | chr07:110,738,836 | *LRRN3;IMMP2L* | -1 |
| 79 | cg21322436 | chr07:145,812,843 | *CNTUnknownP2* | -1 |
| 80 | cg16254309 | chr07:145,814,153 | *CNTUnknownP2* | -1 |
| 81 | cg25949550 | chr07:145,814,306 | *CNTUnknownP2* | -1 |
| 82 | cg11207515 | chr07:146,904,206 | *CNTUnknownP2* | +1 |
| 83 | cg17372101 | chr07:147,500,722 | *CNTUnknownP2* | +1 |
| 84 | cg19717773 | chr07:2,847,554 | *GUnknown12* | -1 |
| 85 | cg02451831 | chr07:26,578,099 | *KIAA0087* | -1 |
| 86 | cg08396193 | chr07:27,193,709 | *HOXA7* | -1 |
| 87 | cg22132788 | chr07:45,002,487 | *MYO1G* | +1 |
| 88 | cg12803068 | chr07:45,002,919 | *MYO1G* | +1 |
| 89 | cg07826859 | chr07:45,020,087 | *MYO1G* | -1 |
| 90 | cg03440944 | chr07:45,023,330 | *C7orf40* | -1 |
| **Table S2.** Continued | | | | |
| 91 | cg10190813 | chr07:48,018,531 | *HUS1* | -1 |
| 92 | cg09022230 | chr07:5,457,226 | *TNRC18* | -1 |
| 93 | cg19589396 | chr08:103,937,374 | *Unknown* | -1 |
| 94 | cg25305703 | chr08:128,378,218 | *Unknown* | -1 |
| 95 | cg12075928 | chr08:141,801,307 | *PTK2* | -1 |
| 96 | cg26361535 | chr08:144,576,604 | *ZC3H3* | -1 |
| 97 | cg24540678 | chr08:28,258,603 | *Unknown* | -1 |
| 98 | cg13518625 | chr08:29,522,838 | *Unknown* | -1 |
| 99 | cg01692968 | chr09:108,005,349 | *Unknown* | -1 |
| 100 | cg25953130 | chr10:63,753,550 | *ARID5B* | -1 |
| 101 | cg03450842 | chr10:80,834,947 | *ZMIZ1* | -1 |
| 102 | cg04039799 | chr11:19,745,485 | *UnknownV2* | -1 |
| 103 | cg01744331 | chr11:2,722,358 | *KCNQ1OT1;KCNQ1* | -1 |
| 104 | cg07123182 | chr11:2,722,391 | *KCNQ1OT1;KCNQ1* | -1 |
| 105 | cg16556677 | chr11:2,722,402 | *KCNQ1OT1;KCNQ1* | -1 |
| 106 | cg26963277 | chr11:2,722,408 | *KCNQ1OT1;KCNQ1* | -1 |
| 107 | cg16611234 | chr11:58,870,075 | *Unknown* | -1 |
| 108 | cg19254163 | chr11:60,623,783 | *GPR44* | -1 |
| 109 | cg21611682 | chr11:68,138,269 | *LRP5* | -1 |
| 110 | cg14624207 | chr11:68,142,198 | *LRP5* | -1 |
| 111 | cg01901332 | chr11:75,031,055 | *ARRB1* | -1 |
| 112 | cg11660018 | chr11:86,510,915 | *PRSS23* | -1 |
| 113 | cg23771366 | chr11:86,510,999 | *PRSS23* | -1 |
| 114 | cg07986378 | chr12:11,898,285 | *ETV6* | -1 |
| 115 | cg02583484 | chr12:54,677,008 | *HNRNPA1;HNRPA1L-2* | -1 |
| 116 | cg04158018 | chr12:54,696,211 | *NFE2* | -1 |
| 117 | cg23681440 | chr13:27,498,239 | *Unknown* | -1 |
| 118 | cg01208318 | chr14:106,329,652 | *Unknown* | -1 |
| 119 | cg01731783 | chr14:74,211,789 | *C14orf43* | -1 |
| 120 | cg22851561 | chr14:74,214,183 | *C14orf43* | -1 |
| 121 | cg24996979 | chr14:74,223,355 | *C14orf43* | -1 |
| 122 | cg10919522 | chr14:74,227,441 | *C14orf43* | -1 |
| 123 | cg13976502 | chr14:74,227,875 | *C14orf43* | -1 |
| 124 | cg13038618 | chr14:77,467,391 | *Unknown* | -1 |
| 125 | cg05284742 | chr14:93,552,129 | *ITPK1* | -1 |
| 126 | cg25292882 | chr15:39,431,467 | *Unknown* | -1 |
| 127 | cg00310412 | chr15:74,724,919 | *SEMA7A* | -1 |
| 128 | cg11152412 | chr15:74,927,688 | *EDC3* | -1 |
| 129 | cg23161492 | chr15:90,357,203 | *ANPEP* | -1 |
| 130 | cg16794579 | chr16:17,562,419 | *XYLT1* | -1 |
| 131 | cg09099830 | chr16:30,485,486 | *ITGAL* | -1 |
| 132 | cg04716530 | chr16:30,485,684 | *ITGAL* | -1 |
| 133 | cg06972908 | chr16:30,488,321 | *ITGAL* | -1 |
| 134 | cg13500388 | chr16:67,062,135 | *CBFB* | -1 |
| 135 | cg19572487 | chr17:38,476,025 | *RARA* | -1 |
| 136 | cg07465627 | chr17:53,167,407 | *STXBP4* | -1 |
| **Table S2.** Continued | | | | |
| 137 | cg07251887 | chr17:73,641,810 | *LOC100130933;RECQL5* | -1 |
| 138 | cg00073090 | chr19:1,265,879 | *Unknown* | -1 |
| 139 | cg03636183 | chr19:17,000,586 | *F2RL3* | -1 |
| 140 | cg15159987 | chr19:17,003,890 | *CPAMD8* | -1 |
| 141 | cg23973524 | chr19:18,873,223 | *CRTC1* | +1 |
| 142 | cg15187398 | chr19:2,093,896 | *MOBKL2A* | -1 |
| 143 | cg07381806 | chr19:2,094,327 | *MOBKL2A* | -1 |
| 144 | cg03707168 | chr19:49,379,127 | *PPP1R15A* | -1 |
| 145 | cg01500140 | chr19:51,890,586 | *LIM2* | +1 |
| 146 | cg16201146 | chr20:19,191,527 | *Unknown* | -1 |
| 147 | cg07339236 | chr20:50,312,491 | *ATP9A* | -1 |
| 148 | cg06595162 | chr21:40,141,041 | *NCRUnknown00114* | -1 |
| 149 | cg23110422 | chr21:40,182,073 | *ETS2* | -1 |
| 150 | cg02532700 | chr22:37,257,404 | *NCF4* | -1 |
| 151 | cg01127300 | chr22:38,614,796 | *Unknown* | -1 |

* weight was substituted as +1 if the CpG is hypermethylated in never smokers and as -1 if the CpG is hypomethylated [14].

| **Table S3.** Confusion matrix for GRS, MRS, pack-years and their combination in LC risk prediction | | | | | | | | | | |
| --- | --- | --- | --- | --- | --- | --- | --- | --- | --- | --- |
| **Predictor** | **Prediction*** | **All participants**** | | | |  | **Heavy smokers** | | | |
|  |  | **Case (n=128)** | **Control (n=1323)** | **Sensitivity** | **Precision** |  | **Case (n=69)** | **Control (n=205)** | **Sensitivity** | **Precision** |
| GRS | + | 39 | 256 | 30% | 13% |  | 20 | 41 | 29% | 33% |
|  | - | 89 | 1058 |  |  |  | 49 | 164 |  |  |
| MRS | + | 80 | 256 | 63% | 24% |  | 21 | 41 | 30% | 34% |
|  | - | 48 | 1058 |  |  |  | 48 | 164 |  |  |
| GRS+MRS | + | 80 | 256 | 63% | 24% |  | 19 | 41 | 28% | 32% |
|  | - | 48 | 1058 |  |  |  | 50 | 164 |  |  |
| Pack-years | + | 81 | 256 | 63% | 24% |  | 21 | 41 | 30% | 34% |
|  | - | 47 | 1058 |  |  |  | 48 | 164 |  |  |
| Pack-years+GRS+MRS | + | 86 | 256 | 67% | 25% |  | 27 | 41 | 39% | 40% |
|  | - | 42 | 1058 |  |  |  | 42 | 164 |  |  |

^*^ cutoff point was derived at specificity of 0.80; + represents a positive prediction and – represents a negative prediction.

^**^ participants with GRS, MRS and pack-years available.

**B**

**A**


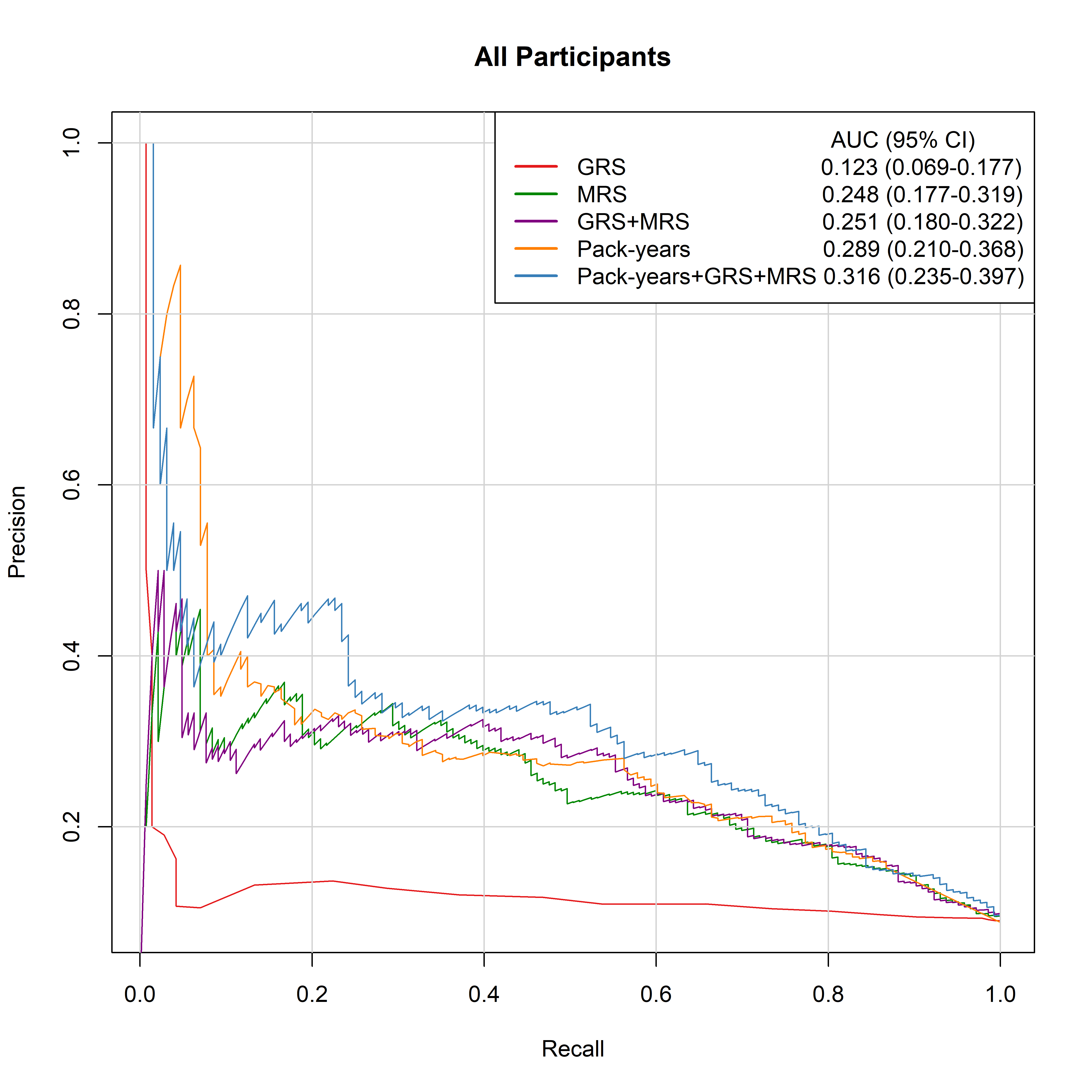

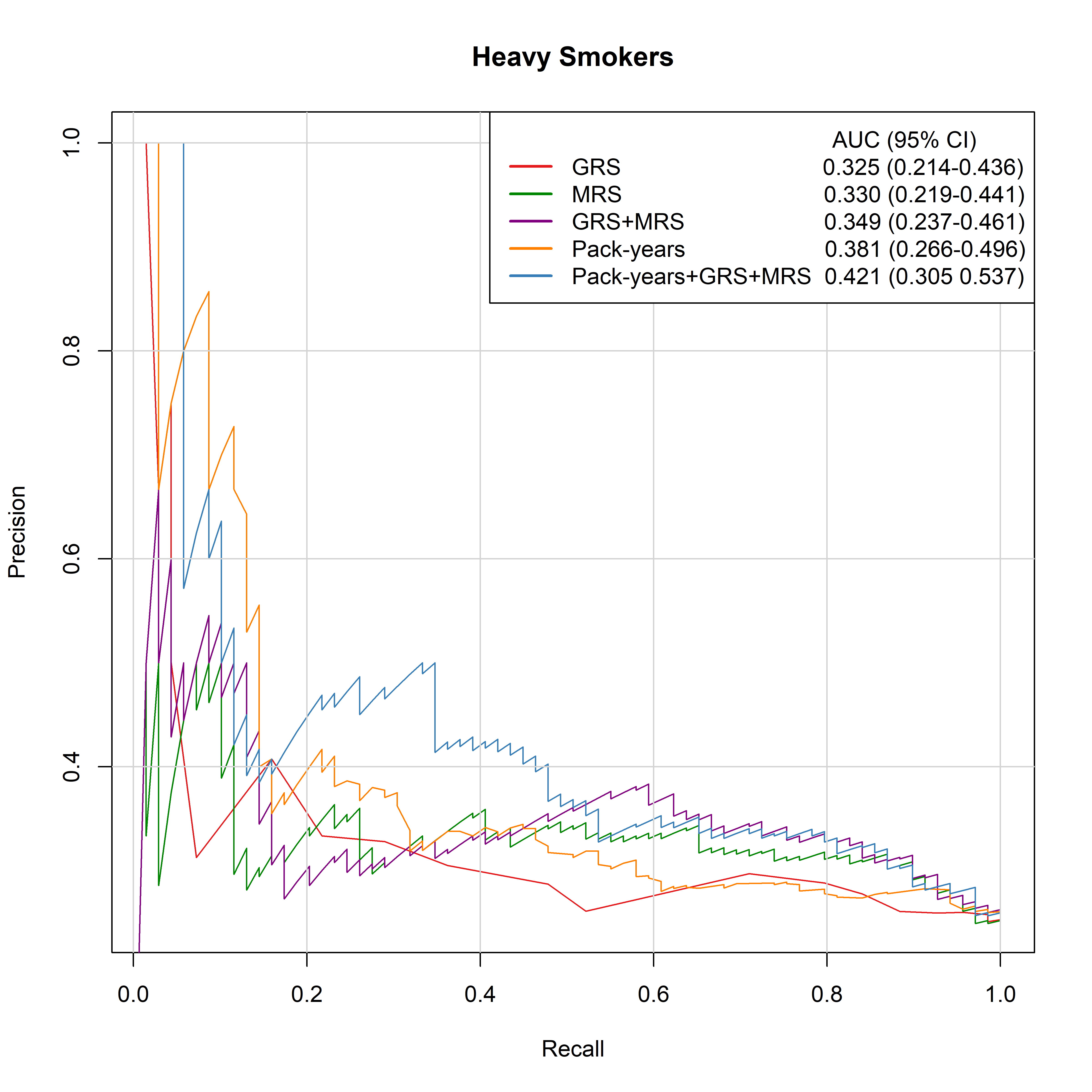


**Figure S1.** Precision-Recall Curves for GRS, MRS, pack-years and their combination in prediction of LC incidence. A. PRC curves for all participants; B. PRC curves for heavy smokers.

**Supplemental references**

1. Bosse Y, Amos CI: **A Decade of GWAS Results in Lung Cancer**. *Cancer Epidemiol Biomarkers Prev* 2018, **27**(4):363-379.

2. McKay JD, Hung RJ, Han Y, Zong X, Carreras-Torres R, Christiani DC, Caporaso NE, Johansson M, Xiao X, Li Y *et al*: **Large-scale association analysis identifies new lung cancer susceptibility loci and heterogeneity in genetic susceptibility across histological subtypes**. *Nat Genet* 2017, **49**(7):1126-1132.

3. Timofeeva MN, Hung RJ, Rafnar T, Christiani DC, Field JK, Bickeboller H, Risch A, McKay JD, Wang YF, Dai JC *et al*: **Influence of common genetic variation on lung cancer risk: meta-analysis of 14 900 cases and 29 485 controls**. *Hum Mol Genet* 2012, **21**(22):4980-4995.

4. Wang YF, Mckay JD, Rafnar T, Wang ZM, Timofeeva MN, Broderick P, Zong XC, Laplana M, Wei YY, Han YH *et al*: **Rare variants of large effect in BRCA2 and CHEK2 affect risk of lung cancer**. *Nat Genet* 2014, **46**(7):736-741.

5. McKay JD, Hung RJ, Gaborieau V, Boffetta P, Chabrier A, Byrnes G, Zaridze D, Mukeria A, Szeszenia-Dabrowska N, Lissowska J *et al*: **Lung cancer susceptibility locus at 5p15.33**. *Nat Genet* 2008, **40**(12):1404-1406.

6. Landi MT, Chatterjee N, Yu K, Goldin LR, Goldstein AM, Rotunno M, Mirabello L, Jacobs K, Wheeler W, Yeager M *et al*: **A Genome-wide Association Study of Lung Cancer Identifies a Region of Chromosome 5p15 Associated with Risk for Adenocarcinoma**. *Am J Hum Genet* 2009, **85**(5):679-691.

7. Wang YF, Broderick P, Webb E, Wu XF, Vijayakrishnan J, Matakidou A, Qureshi M, Dong Q, Gu XJ, Chen WV *et al*: **Common 5p15.33 and 6p21.33 variants influence lung cancer risk**. *Nat Genet* 2008, **40**(12):1407-1409.

8. Broderick P, Wang YF, Vijayakrishnan J, Matakidou A, Spitz MR, Eisen T, Amos CI, Houlston RS: **Deciphering the Impact of Common Genetic Variation on Lung Cancer Risk: A Genome-Wide Association Study**. *Cancer Res* 2009, **69**(16):6633-6641.

9. Hung RJ, Mckay JD, Gaborieau V, Boffetta P, Hashibe M, Zaridze D, Mukeria A, Szeszenia-Dabrowska N, Lissowska J, Rudnai P *et al*: **A susceptibility locus for lung cancer maps to nicotinic acetylcholine receptor subunit genes on 15q25**. *Nature* 2008, **452**(7187):633-637.

10. Thorgeirsson TE, Geller F, Sulem P, Rafnar T, Wiste A, Magnusson KP, Manolescu A, Thorleifsson G, Stefansson H, Ingason A *et al*: **A variant associated with nicotine dependence, lung cancer and peripheral arterial disease**. *Nature* 2008, **452**(7187):638-U639.

11. Amos CI, Wu XF, Broderick P, Gorlov IP, Gu J, Eisen T, Dong Q, Zhang Q, Gu XJ, Vijayakrishnan J *et al*: **Genome-wide association scan of tag SNPs identifies a susceptibility locus for lung cancer at 15q25.1**. *Nat Genet* 2008, **40**(5):616-622.

12. Liu PY, Vikis HG, Wang DL, Lu Y, Wang Y, Schwartz AG, Pinney SM, Yang P, de Andrade M, Petersen GM *et al*: **Familial aggregation of common sequence variants on 15q24-25.1 in lung cancer**. *Jnci-J Natl Cancer I* 2008, **100**(18):1326-1330.

13. Gao X, Jia M, Zhang Y, Breitling LP, Brenner H: **DNA methylation changes of whole blood cells in response to active smoking exposure in adults: a systematic review of DNA methylation studies**. *Clin Epigenetics* 2015, **7**:113.

14. Joehanes R, Just AC, Marioni RE, Pilling LC, Reynolds LM, Mandaviya PR, Guan W, Xu T, Elks CE, Aslibekyan S *et al*: **Epigenetic Signatures of Cigarette Smoking**. *Circ Cardiovasc Genet* 2016, **9**(5):436-447.
